# The cost of community-managed viral respiratory illnesses in a cohort of healthy preschool-aged children

**DOI:** 10.1186/1465-9921-9-11

**Published:** 2008-01-24

**Authors:** Stephen B Lambert, Kelly M Allen, Robert C Carter, Terence M Nolan

**Affiliations:** 1 Vaccine and Immunisation Research Group, Murdoch Children's Research Institute, Royal Children's Hospital, and School of Population Health, University of Melbourne, Melbourne, Victoria, Australia; 2 Queensland Paediatric Infectious Diseases Laboratory, Sir Albert Sakzewski Virus Research Centre, Royal Children's Hospital, and Clinical Medical Virology Centre, University of Queensland, Brisbane, Queensland, Australia; 3 Centre for Clinical Effectiveness Southern Health and Monash Institute of Health Services Research, Monash University, Melbourne, Victoria, Australia; 4 Centre for Health Policy, Programs and Economics, School of Population Health, University of Melbourne, Melbourne, Victoria, Australia; 5 Health Economics Unit, School of Health & Social Development, Deakin University, Burwood, Victoria, Australia

## Abstract

**Background:**

Acute respiratory illnesses (ARIs) during childhood are often caused by respiratory viruses, result in significant morbidity, and have associated costs for families and society. Despite their ubiquity, there is a lack of interdisciplinary epidemiologic and economic research that has collected primary impact data, particularly associated with indirect costs, from families during ARIs in children.

**Methods:**

We conducted a 12-month cohort study in 234 preschool children with impact diary recording and PCR testing of nose-throat swabs for viruses during an ARI. We used applied values to estimate a virus-specific mean cost of ARIs.

**Results:**

Impact diaries were available for 72% (523/725) of community-managed illnesses between January 2003 and January 2004. The mean cost of ARIs was AU$309 (95% confidence interval $263 to $354). Influenza illnesses had a mean cost of $904, compared with RSV, $304, the next most expensive single-virus illness, although confidence intervals overlapped. Mean carer time away from usual activity per day was two hours for influenza ARIs and between 30 and 45 minutes for all other ARI categories.

**Conclusion:**

From a societal perspective, community-managed ARIs are a significant cost burden on families and society. The point estimate of the mean cost of community-managed influenza illnesses in healthy preschool aged children is three times greater than those illnesses caused by RSV and other respiratory viruses. Indirect costs, particularly carer time away from usual activity, are the key cost drivers for ARIs in children. The use of parent-collected specimens may enhance ARI surveillance and reduce any potential Hawthorne effect caused by compliance with study procedures. These findings reinforce the need for further integrated epidemiologic and economic research of ARIs in children to allow for comprehensive cost-effectiveness assessments of preventive and therapeutic options.

## Background

Respiratory virus infections are a major cause of morbidity and healthcare usage in children, resulting in substantial costs for families and society [1-5]. Given their ubiquity, there has been surprisingly little research examining the costs associated with childhood respiratory infections that has involved collecting primary data from families. Even for influenza, the most studied of all respiratory viruses, cost-of-illness and vaccine cost-effectiveness evaluations in children have tended to rely on assumptions or use retrospectively collected estimates, often from surveys, for resource utilisation, such as carer time away from work in seeking healthcare or caring for an ill child [6-9].

There are three pieces of evidence required by those developing health policy in assessing whether to recommend or implement a publicly-funded vaccination program, or any intervention, against respiratory viruses: epidemiology of the targeted illness, the efficacy of the intervention, and the cost-effectiveness of the intervention [10]. All interventions to prevent or treat infections will be associated with a cost of implementation, but cost-effectiveness is determined not only by the cost of the intervention, but also by costs arising from the illness. Getting these data for respiratory viruses, particularly information on indirect costs incurred by families, requires a conjunction of epidemiologic and economic research [11].

The prospect of new and improved influenza vaccines [12], the hope of new vaccines against other respiratory viruses [13], development of novel therapeutic possibilities [14], and the possible use of nonpharmaceutical interventions to contain virus transmission [15-18] all underline the need to more critically weigh the costs and benefits of prevention and treatment for common respiratory tract viruses.

We present here findings from a community-based cohort study using parent-collected specimens for etiologic assignment and diary recording of impact data. These data have been used to calculate virus-specific costs of illness from a societal perspective, including often neglected indirect costs.

## Methods

### The study cohort and acute respiratory illness surveillance

Details of recruitment, composition, and maintenance of the dynamic study cohort have been published elsewhere [19]. Ethics approval for the study was given by the Royal Children's Hospital Ethics in Human Research Committee, Melbourne, and written informed consent was obtained from parents before participation.

This dynamic cohort consisted of one healthy child less than five years of age at time of recruitment from each study family. Children involved in this study were recruited from a number of sources. In Victoria, Australia, maternal and child health nurses (MCHNs) provide support to families during the early childhood years, particularly on issues to do with general health and vaccination. Based on a model used by our group for community vaccine studies [20], MCHNs from 26 local councils assisted with recruitment by providing study information to parents of eligible children. Advertising material for the study was placed in child care and playgroup centers and, because of proximity, we also used bulletin boards and staff e-mail lists at the Royal Children's and the Royal Women's Hospitals in Melbourne. Details about the study child and household demographics were collected at an enrolment home visit, including annual gross household income collected in 2003/2004 Australian dollar values (AUD$). Income was separated into four bands, roughly dividing the study households into quartiles: band 1, less than $52,000 (24% of study households); band 2, $52,000 to $77,999 (28%); band 3, $78,000 to $103,999 (23%); and band 4, $104,000 or greater (25%). The approximate proportions for Australian households during the same period were: band 1, 54%, band 2, 20%, band 3, 13%, and band 4, 13% [21].

Parents undertook daily respiratory symptom surveillance of the study child using a diary card and collected a combined nose-throat swab (NTS) and completed a summary impact diary when the child had an acute respiratory illness (ARI). For this study we used a sensitive ARI definition that had previously been used in an influenza vaccine efficacy study [22] and our pilot study [23,24]. Symptoms were classified as category A (fever, wheezing, shortness of breath, pulmonary congestion or moist cough, pneumonia, or ear infection) and category B (runny nose or nasal congestion, sore throat, cough, muscle aches, chills, headache, irritability, decreased activity or lethargy or weakness, or vomiting). An ARI of interest required one category A or two category B symptoms on a single day [22]. Other than pneumonia, which we asked parents to record only if supported by a health care professional's diagnosis, no illness or symptom details, including a report of otitis media, were validated by study staff or health care professionals. A new ARI could not commence unless there were three symptom free days since the end of the previous ARI. This meant an ARI could contain no more than two consecutive symptom-free days. Study families were asked to continue normal healthcare seeking behaviour and treatments, and were not alerted about the start of the influenza season or asked to alter surveillance during the winter season. Pre-stamped envelopes were provided and families were asked to return all completed study documents (daily symptom diary, impact diaries) at the end of each month. ARI duration was calculated using symptom diary data and ARIs were classified by study staff as being simple (no fever or otitis media recorded), or occurring with fever, otitis media, or with both fever and otitis media [22-24].

The NTS was couriered to the Victorian Infectious Diseases Reference Laboratory (VIDRL) where it was tested for a number of common respiratory viruses using a polymerase chain reaction (PCR) method for adenoviruses and reverse transcription (RT) PCR for RNA viruses: influenza A virus, influenza B virus, respiratory syncytial virus (RSV), parainfluenza viruses I, II, and III (PIVs), and picornaviruses [19]. A letter outlining these test results was sent to parents when these details became available. At completion of the study all specimens were transported to the Queensland Paediatric Infectious Diseases (Qpid) Laboratory where they were tested for human metapneumovirus (hMPV) and human coronavirus NL63 (hCoV-NL63) using RT-PCR [19].

### Impact diary completion

A summary impact diary was used to collect details of resources used during the study child's ARI and was based on an impact diary used in a pilot study [23,24], with some simplification. The units of resource use requested were:

▪ health care visits: number and timing of primary care (general practice) visits, hospital presentations and admissions, and visits to other providers (such as naturopaths, homeopaths);

▪ use of prescribed antibiotics;

▪ laboratory tests performed to investigate the illness;

▪ carer time consumed during the illness seeking health care; and

▪ excess carer time during the illness spent caring for the ill child.

We did not collect information about some items that were shown not to be major cost drivers in the pilot study: non-antibiotic prescription medication, over-the-counter and other medication, paid childcare for other children whilst normal carers were spending time caring for the ill study subject, and travel costs seeking health care. The average total cost for these items in the pilot study [23] was AUD$16 per ARI.

Time values were captured in hours and minutes. Parents were not given instructions about when or how frequently they should capture time data during an ARI. For both carer time spent seeking healthcare and excess time spent caring for an ill child, time was recorded as a total value for the ARI in two categories: time away from work and time away from usual, non-work activities.

### Costing methods

All costs were incurred over a 380 day period between 17 January 2003 and 31 January 2004. Costs are reported in this manuscript using Australian dollar values, with 2003 taken as the reference year for reporting unit prices. The mean exchange rates for major currencies during the study were: United Kingdom (UK) pound £1 = AUD$2.49, Euro €1 = AUD$1.73, and United States (US) $1 = AUD$1.50 [25]. Discounting costs for time preference is not routinely considered for periods of time less than 12 months, and as this study period barely exceeds this time frame, no costs have been discounted.

Details of the source and value for all costs are provided (Table 1). Applied costs were retrieved, where possible, from published sources, and where no standard published cost was available we used costs derived from the pilot study. Resource costs were allocated as being borne by either the 'patient and family' sector, the 'healthcare' sector, or the 'employer' of absent staff. The proportions of time away from work seeking healthcare or time away from work caring for an ill child that were incurred by either the patient and family sector or met by an employer were not collected, and these values have been derived from the same proportions in the pilot study, based on 202 illnesses (Table 1) [23].

**Table 1:** Source, sector distribution, and value of applied costs used in costing calculations for acute respiratory illnesses

Resource item	Sector	Source of applied cost	Applied cost	Value
Primary care visits	Patient and family	Medicare Australia [60,61]	Mean patient contribution per service by quarter, patient and bulk billed services from general practitioners and vocationally registered general practitioners	January to March 2003: $4.03 April to June 2003: $4.08 July to September 2003: $4.34 October to December 2003: $4.61 January 2004: $4.64
	Health	Medicare Australia [62,63]	85% (reimbursable amount) of code 23, Medicare Benefits Schedule	January to October 2003: $25.05 November 2003 to January 2004: $25.70

Specialist visits	Patient and family	Medicare Australia [60,61]	Mean patient contribution per specialist visit by quarter	January to March 2003: $19.30 April to June 2003: $19.56 July to September 2003: $19.74 October to December 2003: $20.36 January 2004: $21.65
	Health	Medicare Australia [62,63]	85% (reimbursable amount) of code 104, Medicare Benefits Schedule	January to October 2003: $58.95 November 2003 to January 2004: $60.45

Other health care provider visits	Patient and family	Pilot study [23]	Mean other health care provider visit cost from pilot study, derived from 10 visits	$15.63 per visit to allied and alternative health professionals

Hospital emergency department visits without admission	Health	The Australian Government Department of Health and Ageing [64]	Australian Ambulatory Classes group 23 (other respiratory diseases without procedure)	$40 per visit

Diagnostic tests	15% Patient and family 85% Health	Medicare Australia [62,63]	Medicare Benefits Scheduled fee for diagnostic tests	January 2003 to January 2004 $28.35 Chest x-ray (code 58500) January to October 2003 $14.20 Full blood examination (code 65070) $16.35 Urea, electrolytes, creatinine, liver function tests (code 66515) $17.10 Urine microscopy, culture, identification, and sensitivity (code 69333) November 2003 to January 2004 $14.65 Full blood examination (code 65070) $16.85 Urea, electrolytes, creatinine, liver function tests (code 66515) $17.60 Urine microscopy, culture, identification, and sensitivity (code 69333)

Antibiotics	Patient and family	Pilot study [23]	Mean antibiotic course cost from pilot study, derived from 42 courses	$13.80 per course

Time seeking health care away from work	61% Patient and family 39% Employer	Australian Bureau of Statistics [26]	Average weekly, full-time adult total earnings for females and males used to provide an hourly rate (based on a 38 hour working week)	January 2003 Female $21.62 Male $26.67 February to April 2003 Female $21.94 Male $27.10 May to July 2003 Female $22.25 Male $27.51 August to October 2003 Female $22.49 Male $27.81 November 2003 to January 2004 Female $22.71 Male $28.00
			
Time seeking health care away from usual activity	Patient and family			
			
Excess time caring for ill child away from work	31% Patient and family 69% Employer			
			
Excess time caring for ill child away from usual activity	Patient and family			

Hospitalization	Health	Public hospital admission National Hospital Cost Data Collection [65,66]	Victorian values for code E62C (Respiratory infection/inflammation without complication or co-morbidity)	Round 7 (2002/2003) $2,331 Round 8 (2003/2004) $2,441

We applied a sex-weighted hourly wage rate derived from the Australian Bureau of Statistics average weekly full-time adult total earnings for all reported times [26]. We calculated mean costs (total and by categories) with 95% confidence intervals (95% CI) and median costs with interquartile ranges for ARIs in study children. Data were analysed using Stata 9.2 for Windows (StataCorp, Texas, USA).

## Results

There were 234 children, one from each study family, progressively enrolled in the study and we identified 730 ARIs in 56,397 child-days of follow-up [19]. Of these, 487 ARIs (67%) had at least one specimen and an impact diary available, 41 (6%) had an impact diary returned but no specimen, 56 (8%) had at least one specimen returned but no impact diary available, and 146 (20%) had neither a specimen nor impact diary returned. Children aged between one and two-years of age contributed the most person-time to the study (28% of all child-days) and had the highest acute respiratory illness (ARI) incidence rate (0.56 ARIs per child-month). Contribution by males and females was equivalent, and children who attended some form of out-of-home care were responsible for 67% of all person-time [19].

There were five illnesses which involved a hospital admission, all with an impact diary available. The mean cost of these five ARIs, including the cost of admission, was $3,409 (95% CI $2,798 to $4,020). Of the remaining 725 ARIs, the 202 illnesses without an impact diary had a mean duration of 8.8 days compared with 13.5 days for ARIs with impact data available; median 6 days versus 11 days. Simple ARIs made up 57% (116/202) of no impact diary ARIs and 47% (248/523) of ARIs with an impact diary.

The 523 illnesses with a diary returned that did not involve a hospital admission had a total cost of $161,454 (Table 2), and mean cost of $309 (95% CI $263 to $354). As our particular interest is in the cost of community-managed ARIs, that is, those illnesses that do not require an admission to hospital, further analyses will include only these 523 illnesses.

**Table 2:** Total cost, mean cost (95% confidence interval), and median cost (interquartile range) of acute respiratory illnesses by virus identification

	Number (%)	Total cost	Mean cost	95% confidence interval	Median cost	Interquartile range
Influenza A virus	17 (3.3%)	$15,366	$904	$89 to $1719	$571	$162, $1023
RSV^a^	33 (6.3%)	$10,047	$304	$194 to $415	$198	$60, $398
Picornaviruses	197 (37.7%)	$52,597	$267	$211 to $323	$124	$32, $337
hCoV-NL63^b^	6 (1.1%)	$1,508	$251	-$77 to $580	$83	$30, $625
Adenoviruses	17 (3.3%)	$4,212	$248	$140 to $356	$185	$90, $341
PIVs^c^	21 (4.0%)	$4,804	$229	$104 to $354	$112	$84, $291
hMPV^d^	15 (2.9%)	$3,284	$219	$109 to $328	$204	$57, $324
Co-identifications	51 (9.8%)	$17,503	$343	$212 to $475	$185	$72, $431
No virus identified	126 (24.1%)	$39,853	$316	$208 to $425	$151	$44, $364
No specimen	40 (7.6%)	$12,281	$307	$212 to $402	$216	$88, $434
All ARIs^e^	523 (100.0%)	$161,454^f^	$309	$263 to $354	$156	$45, $378

^a ^Respiratory syncytial virus.

^b ^Human coronavirus NL63.

^c ^Parainfluenza viruses.

^d ^Human metapneumovirus.

^e ^Acute respiratory illnesses.

^f ^Column total does not equal column sum due to rounding.

There were 248 simple ARIs (ARIs without fever or otitis media), with a mean cost of $180 (95% CI $131 to $230). The 207 ARIs with fever had a mean cost $406 (95% CI $318 to $494), the 26 ARIs with otitis media had a mean cost $362 (95% CI $203 to $520), and the 42 ARIs with fever and otitis media had a mean cost of $553 (95% CI $395 to $711). By household income band, there were 121 ARIs from the lowest band (ARI incidence rate: 0.35 per child-month) with a mean cost of $222 (95% CI $174 to $270), 144 ARIs from band 2 (0.37 ARIs per child-month) had a mean cost $375 (95% CI $244 to $506), 110 ARIs from band 3 (0.44 ARIs per child-month) with mean cost $366 (95% CI $282 to $451), and 148 ARIs from band 4 (0.43 ARIs per child-month) with mean cost $272 (95% CI $208 to $337). The mean cost of an ARI in a female subject was $67 greater than their male counterparts: female mean cost $341 (95% CI $265 to $418), male mean cost $274 (95% CI $228 to $319).

The mean and median costs by virus identification, including co-identification and specimen availability, are provided (Table 2). The differences between the mean values and the median values demonstrate the right-skewed nature of these data, similar to other health-related costs [27]. Whilst confidence intervals overlap, the point estimate of the mean cost of an influenza A ARI, $904, is three times higher than the next most expensive single virus ARI: RSV $304. Of the 51 ARIs where more than one virus was identified, influenza A virus was present in four: two illnesses with co-identification with a picornavirus alone, one illness with hCoV-NL63 alone, and one illness with a picornavirus and PIV. These four illnesses had a mean cost of $499. There were no illnesses where influenza B virus was identified. Three children had received influenza vaccine in the year prior to the study and none had an influenza-positive ARI.

As the difference in mean cost between the most expensive (RSV: $304) and least expensive (hMPV: $219) non-influenza single virus ARI falls within a comparatively narrow band ($85) we collapsed these data into a single category for further comparisons (Table 3). The mean cost for non-influenza single virus ARIs was $265 (95% CI $223 to $306). The cost of excess carer time away from a usual activity averaged $706 per ARI for influenza A, making up 78% of the total mean cost of illness, compared with $164 and 62% for other single virus illnesses.

**Table 3:** Mean values and mean cost of components of resource use during ARIs

	Number of ARIs	Primary care visits		All other healthcare costs^a^	Seeking healthcare time off work^b^			Seeking healthcare time away from usual activity^b^			Excess care time off work^b^			Excess care time away from usual activity^b^		
		Mean number	Mean cost	Mean cost	Mean male time	Mean female time	Mean cost	Mean male time	Mean female time	Mean cost	Mean male time	Mean female time	Mean cost	Mean male time	Mean female time	Mean cost

Influenza A virus	17	1.11	$32.85	$12.84	0.00	0.12	$2.65	0.12	0.94	$24.37	1.76	3.38	$125.16	4.29	26.09	$706.00
Other single virus	289	0.57	$16.73	$7.19	0.02	0.55	$12.83	0.04	0.66	$15.64	0.47	1.60	$48.55	1.09	6.01	$163.61
Co-identifications	51	0.76	$22.46	$3.79	0.00	0.38	$8.43	0.03	0.50	$11.90	1.49	2.49	$96.41	1.46	7.18	$200.20
No virus identified	126	0.63	$18.65	$7.07	0.02	0.22	$5.58	0.03	0.67	$15.79	0.88	1.60	$60.23	1.48	7.53	$208.97
No specimen returned	40	0.80	$23.72	$25.70	0.00	0.48	$10.64	0.41	1.26	$39.66	0.69	1.81	$59.31	1.13	5.25	$148.01
All ARIs^c^	523	0.64	$18.81	$8.43	0.02	0.44	$10.15	0.07	0.70	$17.43	0.73	1.76	$59.34	1.33	7.08	$194.54

^a ^All other healthcare costs is the sum of hospital emergency department presentations, other provider costs (specialists, other therapists), laboratory tests, and prescribed antibiotics.

^b ^All time recorded in hours.

^c ^Acute respiratory illnesses.

Of the mean cost for all illnesses, $19 (6%) was met by the healthcare sector, $245 (79%) by the patient and family sector, and $45 (15%) was met by employers paying for an employee who was seeking healthcare for or caring for an ill child. The equivalent values for influenza A infections are: $36 (4%), $780 (86%), and $87 (10%), respectively. In influenza A ARIs the key cost driver, carer time away from usual activity, resulted in a mean loss of two hours per day per illness. For all other illness categories, this value ranged from one half to three quarters of an hour per day per ARI.

There was little difference in the mean duration of influenza A illnesses and other single virus illnesses, but co-identifications were 2.2 and 3.6 days longer than each of these respectively (Table 4). The mean delay between illness onset and a result letter being sent was shortest in influenza illnesses at 6.3 days (Table 4).

**Table 4:** Mean duration of ARI and mean delay for result letter

	Mean duration (days)	Mean delay from ARI^a ^onset to results letter being sent (days)
Influenza A virus	15.2	6.3
Other single virus	13.8	8.9
Co-identifications	17.4	8.8
No virus identified	12.2	8.4
No specimen returned	10.5	--
All ARIs^a,b^	13.5	8.7

^a ^Acute respiratory illnesses.

^b ^Includes all ARIs with a specimen returned.

## Discussion

In this study we present the costs associated with community-managed respiratory viral infections in healthy preschool aged children. These costs are based on the direct recording of impact information captured by parents when the study child was unwell. The study has a unique combination of features including a sensitive definition for ARI, parent-collected specimens, laboratory testing for respiratory viruses using sensitive molecular methods, and, based on findings from our pilot study, comprehensive collection of costs, including the previously neglected indirect cost, time away from a usual, non-work activity.

We found, from a societal perspective, the point estimate for the mean cost of all ARIs ($309; 95% CI $263 to $354) was not dissimilar to the mean value we calculated from the pilot study ($241; 95% CI $191 to $291) [23] using the same ARI definition and a slightly modified impact diary. The use of PCR testing for diagnosis on collected specimens allowed us to assign impact and costs to specific viral agents. For all but influenza A illnesses, the cost of community-managed ARIs in healthy preschool-aged children fell within a relatively narrow $85 range. Despite overlapping confidence intervals, the finding of most note in this study was the dramatically higher point estimate of the mean cost of influenza A ARIs, being three times higher than illnesses caused by RSV and the other common respiratory viral infections of childhood. The presence of fever and/or otitis media generally increased the mean cost of illness; but despite having a high prevalence of fever, a longer mean duration, and higher primary care usage [19], adenoviral infections, for example, did not have the cost burden of influenza infections. This highlights the pivotal contribution of excess carer time away from usual non-work activity to total costs, making it the key cost driver for all ARIs in children and differentially amplifying the total costs of influenza illnesses. Whilst the confidence intervals for mean cost of influenza A ARIs and other single virus ARIs overlap, due to the relatively small number of influenza illnesses available for costing, we believe it is unlikely that chance could account for such an extreme difference.

The availability of preventive vaccines and specific therapeutic options makes influenza the most studied of respiratory viruses in all age groups; no other virus is more predictably disruptive year-on-year than annual interpandemic influenza [2-5]. Studies conducted in the second half of last century [28-31] and recent observation [32,33] and intervention [34-36] studies show children have comparatively higher rates of influenza infection and are the most important transmitters of infection within households and communities. Whilst dollar amounts may not directly translate, impact data from this study may be transferable to other countries with developed economies, and similar disease epidemiology and healthcare systems. Ideally further studies in other countries should be conducted to allow for an examination of how impact and cost data vary with the nature of the healthcare system, local virus epidemiology, and other societal factors, including household structure.

Despite lower mean costs than influenza illnesses and the lack of population-based prevention options, the importance of working towards the prevention of other respiratory viral infections is obvious. Picornavirus ARIs, though typically milder and more difficult to be certain of a causal association with illness [37,38], were associated with the highest overall costs of any viral group totalling over $50,000 or one-third of all costs, for the 12-month study period. In the absence of specific vaccines and therapies for other viruses, the application of nonpharmaceutical interventions at a population level, such as improved hand and respiratory hygiene, may have an important place in reducing illness due to respiratory viruses [16].

Our findings reinforce the importance of virus testing in such studies to accurately estimate epidemiology and costs [11]. These data add to accumulating evidence that laboratory confirmation of influenza, in particular, is required, rather than less specific influenza-like illness (ILI) definitions or hospital coding. Other recent studies have found laboratory-confirmed influenza hospitalizations were two to four times more costly [39-41] than shown in previous studies using coding-based estimates [6,42-44]. When ILI definitions or coding are used, rather than laboratory confirmation, a lack of specificity results in influenza illnesses being mixed with other agents, thereby considerably diluting cost differences [45,46]. A direct comparison of parent-collected NTS specimens with collection of a more invasive specimen, such as a nasopharyngeal aspirate, by a healthcare worker at the time of an ARI was beyond the scope of this study. Any reduction in sensitivity caused by the type of specimen used is likely to minor: our finding that 74% of all specimens collected from children in this study were positive for at least one virus is within the range of values from recent home visit studies which also used PCR for diagnosis and nasopharyngeal aspirates (69%) [47] or nasal lavages (83%) [48].

There are clearly some issues about the cost of illnesses caused by respiratory viruses in children unresolved by our study, and some issues that need to be considered before interpretation. Despite being a relatively large cohort the number of illnesses on which to make costing estimates for some virus types is quite small. Further community-based estimates are required to not only confirm our findings but to improve precision around point estimates.

Compared with the Australian population, households with lower incomes were under-represented in our study sample, and, despite overlapping confidence intervals around income band point estimates of mean costs, this may have lead to an overestimation of total costs. However, this may be balanced somewhat by the over-representation of households from the top income band which had a relatively lower mean ARI cost ($272). This pattern of household income distribution was similar to that found in the pilot study [23]. For this study we sought to make our study sample more representative of the general community by focusing our recruitment efforts in local council areas with a higher proportion of lower income households. We have no empiric data available that would allow us to quantify the effect of any potential bias resulting from this skewed sample. Other recent burden studies do not report similar household level income data to allow for comparison [49-51]. It may be the case that lower income households are under-represented as they do not have the spare capacity required, in time or other resources, to allow for study involvement.

We received impact diaries for just over 70% of all ARIs identified by daily symptom surveillance. ARIs without a diary were more likely to be shorter and without fever or otitis media; any information bias resulting from this would likely be in the direction of inflating mean illness costs. Our study only captured information from a single season with higher than normal influenza activity with H3N2 influenza A (drifted strain subtype A/Fujian/411/2002-like) being the predominant circulating type [52]. Variations in incidence and severity year-by-year for all respiratory viruses make it difficult to directly translate our findings to other years.

We believe documenting all time spent on caring for an ill child is important, even when taken away from a usual activity. We appreciate that applying standard wage rates to leisure time is not a straightforward issue in economics. This approach values carer leisure time and non-paid working time in a similar way to a worker's time consistent with neoclassical theories of labour economics [53]. In attaching value to leisure time and using sex-weighted wage rates, we have made our assumptions explicit, and provided sufficient detail (Table 3) so that others can adjust unit prices using different approaches. Previous burden data [49] have been used to assess the cost-effectiveness (C/E) of using influenza vaccine in children [54]. If our cost values, incorporating these indirect costs, were used in the numerator of C/E calculations, there is a distinct possibility of double counting [55]. Double counting is likely where the denominator is a utility measure that incorporates a quality assessment (such as the quality adjusted life year or QALY), and most economists would see leisure time as a logical component of the QALY. There is also debate [53] about the inclusion, measurement, and valuation of lost working time in economic evaluations, with the debate centring on whether in practice QALY instruments capture income effects related to absenteeism.

For all illnesses where a specimen was tested, parents received a result letter by mail. The delay between illness onset and posting the letter was shortest for influenza illnesses, but for most illnesses parents would have been aware of the result before illness end. Pandemic influenza was not being widely discussed in Australia during 2003, but interpandemic influenza does receive media coverage annually encouraging vaccine uptake, and this may have caused parents to overestimate key parameters associated with their child's influenza-positive illness. However, if such a bias was in operation it might also be expected that time values for illnesses where no virus was identified may be relatively understated when compared to ARIs with one or more viruses present. We did not find such a phenomenon; ARIs with no virus identified had a higher mean cost than those with a single virus present, and for the key cost driver of excess carer time away from a usual activity, no cause illnesses had higher values than both single and multiple virus ARIs.

Despite the impact of respiratory viral infections in children there are relatively few burden comparisons available that collect primary data from ill children. An Italian study examining the impact of hMPV, RSV, and influenza in children less than 15-years of age presenting to an emergency department found hMPV illnesses to be significantly more burdensome than RSV, having a similar impact to influenza [50]. In our study hMPV was the least expensive single-virus illness. This finding may be due to the different nature of illnesses that result in hospital presentation or hospital admission, compared with community managed illness. Of the 730 ARIs in this study only 4.0% (n = 29) prompted hospital presentation, with less than 1% (n = 5) requiring admission. An excellent community-based Finnish study describing the burden of influenza in children 13-years of age or younger over two seasons, with 2231 child-seasons of data, also contrasts this imbalance between community-managed and hospitalized cases of influenza, with only three emergency department referrals and one hospital admission in 131 children less than three years of age with influenza [49]. This study differed from ours in that whilst it used laboratory confirmation, it did not employ more sensitive molecular diagnostics [56], families were required to visit the study clinic when the study child had fever or signs of respiratory infection, indirect costs did not include non-work time away from a usual activity, and the study did not provide a comparison with other viral acute respiratory illnesses [49]. The findings from the Finnish study reinforce the need to follow children for ARIs over more than one season, with different rates of influenza infection from year-to-year in each age group. These differences extended to changes in likelihood of infection between age groups: for example, the rate of laboratory-confirmed influenza increased by one-third from season one (2000–2001) to season two (2001–2002) for children less than three years of age, but the rates for three to six year olds and seven to 13 year olds fell 47% and 86%, respectively. A German study, recruiting children less than three years of age with lower respiratory tract infection (LRTI) through office and hospital-based paediatricians, collected cost of illness from a societal perspective, including loss of work days by caregivers [51]. This study showed that non-hospitalized cases of influenza LRTI had twice the cost of PIV LRTI and were one-third more costly than RSV LRTI, with this difference made up entirely by indirect costs [51].

Whilst methods vary, previous cost effectiveness studies of influenza vaccine in children are characterised by two findings: first, that cost-effectiveness is unsurprisingly enhanced by taking a societal perspective through the inclusion of indirect costs [5,6,8,43,54,57]. Our findings reinforce the importance of indirect costs [51], and highlight a previously inadequately measured layer of burden – carer time away from a usual, non-work activity. Second, the potential cost-effectiveness of implementing a vaccination program is improved by flexible or non-individual based delivery programs [6,43]. Vaccine delivered through pharmacies for a small service fee – improving access and negating the time and costs associated with a primary care visit – or large school-based programs, are likely to be acceptable to parents and providers. It is likely that the cost benefits of preventing influenza in children would extend beyond the targeted age-group [58], similar to the indirect effects in older age groups seen following the introduction of childhood conjugate pneumococcal vaccination in the US [59].

## Conclusion

Our study reinforces the costly impact of all respiratory viruses, but particularly interpandemic influenza, on children, their families, and society. Efforts to further explore the costs associated with community-managed illness over a number of seasons for all respiratory infections are needed. Similar to recent hospital-based findings, using laboratory-confirmation to specifically identify influenza appears to increase the cost of illness many fold; a finding that may make population-based vaccination programs a more cost-effective proposition.

We believe the use of parent-collected specimens may have important effects in reducing bias in both the epidemiologic and impact data collected. Not requiring parents to either present with their ill child to a health clinic or host a home visit by study staff may result in enhanced ARI surveillance, but more importantly, allows for the reporting of impact data uncontaminated by compliance with study procedures, thereby reducing any impact a Hawthorne effect may have. Further studies that collect primary, integrated epidemiologic and economic data, particularly indirect costs, directly from families about community-managed ARIs in children, are required. Such data would allow for a more informed exploration of the cost-effectiveness of vaccine programs and other interventions designed to reduce the morbidity associated with ARIs in children.

## Abbreviations

ARI: Acute respiratory illness; AUD: Australian dollars; C/E: Cost effectiveness ratio; CI: Confidence interval; hCoV: Human coronavirus; hMPV: Human metapneumovirus; ILI: Influenza-like illness; LRTI: Lower respiratory tract infection; MCHN: Maternal and child health nurse; NTS: Nose-throat swab; PCR: Polymerase chain reaction; PIV: Parainfluenza virus; QALY: Quality adjusted life year; Qpid: Queensland Paediatric Infectious Diseases; RNA: Ribonucleic acid; RSV: Respiratory syncytial virus; RT: Reverse transcription; UK: United Kingdom; US: United States; VIDRL: Victorian Infectious Diseases Reference Laboratory.

## Competing interests

Terence Nolan and Stephen Lambert have, in the past five years, received research grants for epidemiological and vaccine related research from CSL Limited, Medimmune, GSK Biologicals, Wyeth, and Merck. Kelly Allen and Robert Carter have no competing interests to declare.

## Authors' contributions

All authors were involved in the study design and approach and SBL and TMN developed the original protocol. KMA and SBL were responsible for the day-to-day conduct of the study. SBL performed the analysis and drafted the article. All authors contributed to and approved the final manuscript.
